# *In Silico* and *In Vivo* Anti-Malarial Studies of 18β Glycyrrhetinic Acid from *Glycyrrhiza* *glabra*

**DOI:** 10.1371/journal.pone.0074761

**Published:** 2013-09-24

**Authors:** Komal Kalani, Jyoti Agarwal, Sarfaraz Alam, Feroz Khan, Anirban Pal, Santosh Kumar Srivastava

**Affiliations:** 1 Medicinal Chemistry Department, CSIR-Central Institute of Medicinal and Aromatic Plants, Lucknow, Uttar Pradesh, India; 2 Molecular Bioprospection Department, CSIR-Central Institute of Medicinal and Aromatic Plants, Lucknow, Uttar Pradesh, India; 3 Metabolic and Structural Biology Department, CSIR-Central Institute of Medicinal and Aromatic Plants, Lucknow, Uttar Pradesh, India; Wake Forest University, United States of America

## Abstract

Malaria is one of the most prevailing fatal diseases causing between 1.2 and 2.7 million deaths all over the world each year. Further, development of resistance against the frontline anti-malarial drugs has created an alarming situation, which requires intensive drug discovery to develop new, more effective, affordable and accessible anti-malarial agents possessing novel modes of action. Over the past few years triterpenoids from higher plants have shown a wide range of anti-malarial activities. As a part of our drug discovery program for anti-malarial agents from Indian medicinal plants, roots of *Glycyrrhiza* *glabra* were chemically investigated, which resulted in the isolation and characterization of 18β-glycyrrhetinic acid (GA) as a major constituent. The *in vitro* studies against *P. falciparum* showed significant (IC_50_ 1.69µg/ml) anti-malarial potential for GA. Similarly, the molecular docking studies showed adequate docking (LibDock) score of 71.18 for GA and 131.15 for standard anti-malarial drug chloroquine. Further, *in silico* pharmacokinetic and drug-likeness studies showed that GA possesses drug-like properties. Finally, *in vivo* evaluation showed a dose dependent anti-malarial activity ranging from 68–100% at doses of 62.5–250mg/kg on day 8. To the best of our knowledge this is the first ever report on the anti-malarial potential of GA. Further work on optimization of the anti-malarial lead is under progress.

## Introduction

Malaria is an endemic infection caused by the protozoan parasites belonging to the genus *Plasmodium* [1]. The disease has positioned about 3.3 billion people at risk. The recommended preventive drugs are a combination of sulfadoxine, pyramethamine and amodiaquine, while the therapeutic strategy includes use of artemisinin combinations in areas where *P. falciparum* is endemic and chloroquine (CQ) in the areas where it is still efficacious such as some American regions [2]. This enormous global health challenge is partly due to the development of resistance against the frontline drugs especially artemisinins, which has been recently detected in the four countries of the Asian sub-continent. This alarming situation calls for an intensive drug discovery to find out new natural leads. The resistance in malaria parasites is believed to have emerged through mutations in the active sites of drug targets or from the biochemical changes in the drug receptors [3]. After the successful elucidation of complete genome sequence of *P. falciparum* [4], it has opened a new prospect in the drug discovery where the available databases and bio-informatics tools help us to determine potential receptors and targets within the pathogen [5]. Some of the new drug targets identified in *P. falciparum* are *Plasmodium falciparum* lactate dehydrogenase enzyme (*pf*LDH), type II fatty acid synthase (type II FAS) and plasmepsin. The *pf*LDH has been considered as a potential molecular target for anti-malarials due to this parasite’s dependence on glycolysis for energy production. The *pf*LDH has significantly different structural and kinetic properties compared to human LDH iso-forms [6], but the LDH enzymes found in *P. vivax, P. malariae* and *P. ovale* (pLDH) all exhibit, ~ 90% identity to *pf*LDH and several selective inhibitors of pLDH have demonstrated anti-malarial activity *in-vitro* and *in-vivo*. Similarly, *plasmodium* fatty acids are synthesized in the apicoplast, utilizing a dissociable multi-enzyme system called type II fatty acid synthase (type II FAS), which differs significantly from human type I FAS [7]. On the other hand, plasmepsins are a class of at least 10 enzymes produced by the *P. falciparum* parasite. There are ten different isoforms of these proteins and ten genes coding them respectively in *Plasmodium*. Through their hemoglobin-degrading activity, they are an important cause of symptoms in malaria sufferers. Hence, it would be desirable to use *pf*LDH, type II FAS and plasmepsin as potential targets for the structure-based design of novel anti-malarial.

Recently, CoMFA, CoMSIA, QSAR and docking guided selection of type II FAS, plasmepsin and pLDH inhibitors have been used as important strategies in the discovery of new anti-malarials [3,8,9,10]. These approaches are inexpensive and more practical than discovering novel compounds.

Over the past few years, triterpenoids from higher plants have shown a wide range of biological activities, such as antitumor [11], antiviral [12], anti-inflammatory [13,14,15] and anti-HIV [16]. As a part of our drug discovery program on anti-malarial agents from Indian medicinal plants, the literature search revealed significant anti-malarial activity in pentacyclic triterpenes, betulinic, oleanolic and ursolic acids [17,18]. This prompted us to investigate anti-malarial activity in other triterpenoids, found in widely used Indian medicinal plants. For this purpose, roots of *Glycyrrhiza* *glabra* were taken for detailed chemical and biological investigations. *G* *. glabra* is one of the most important Indian medicinal plants regularly used by common masses from the ancient time. It is used to reduce the toxicity and the bitter taste of other drugs as well as it enhances the effectiveness of other gradients [19]. For more than 4000 years, it has been used against acute bronchitis, severe mouth ulcer, inflammation and other stomach related diseases [19,20].

**Figure 1:**
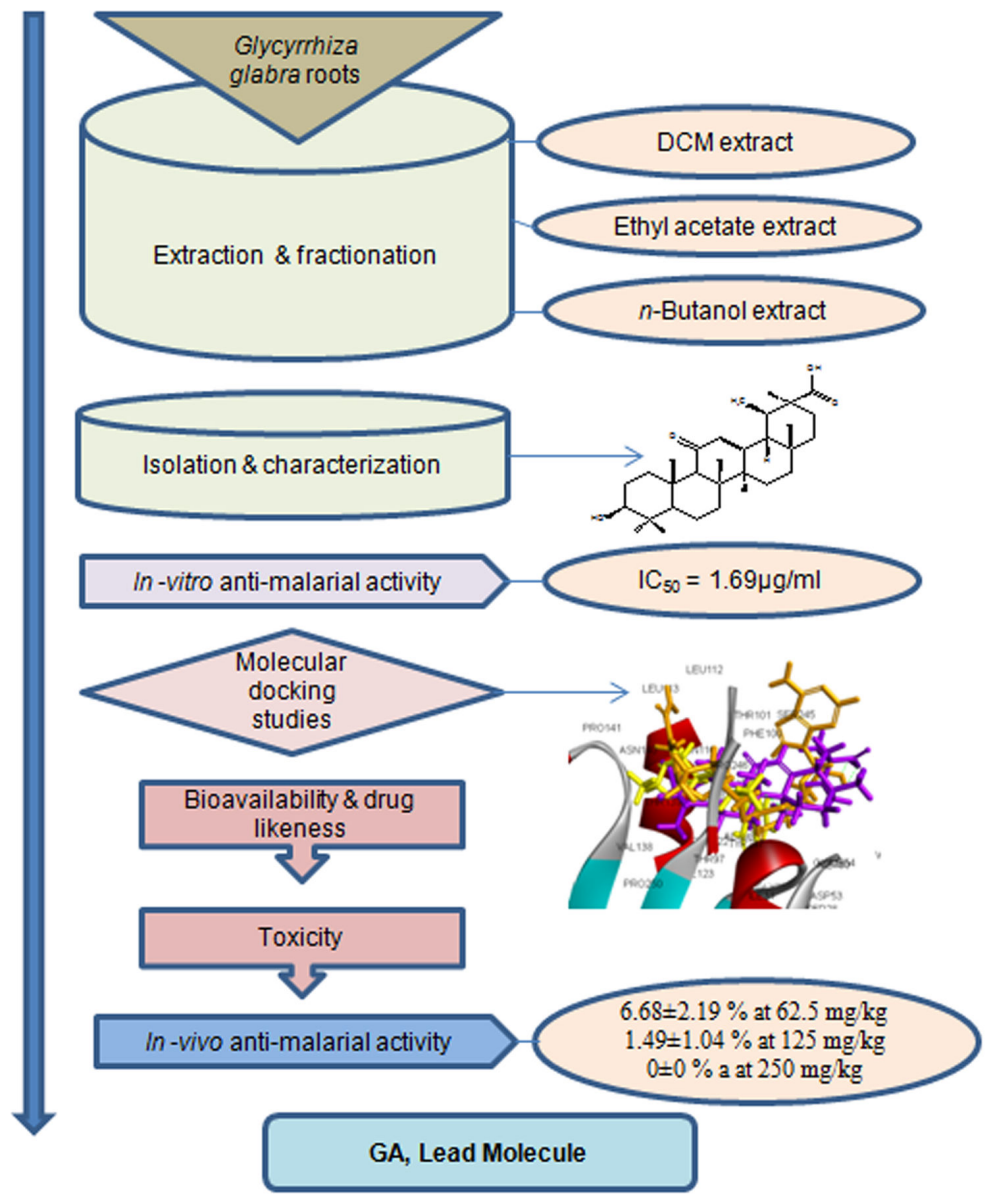
Schematic isolation, identification and bio-evaluation of anti-malarial lead, 18β-glycyrrhetinic acid (GA).

The present study reports isolation and characterization of 18β-glycyrrhetinic acid (GA) as a major constituent from the roots of *G* *. glabra* followed by its *in-vitro, in-silico* and *in vivo* anti-malarial evaluation as shown in the Figure 1.

## Experimental Section

### Ethics statement

The animal experimentation was duly approved by the Institutional Animal Ethics Committee under the Committee for the Purpose of Control and Supervision of Experimentation on Animals, Govt. of India.

### 2.1: Isolation of 18β-glycyrrhetinic acid

#### Plant Material

The roots of *G* *. glabra* were collected after cultivation and harvesting from the experimental farm of Central Institute of Medicinal and Aromatic Plants (CIMAP), Lucknow, Uttar Pradesh, India during the month of January, 2008. A voucher specimen # 9900 was deposited in the Herbarium section of the Botany Department of CIMAP.

#### 2.1.1: Extraction and Fractionation of *G* *. glabra* roots

**Figure 2:**
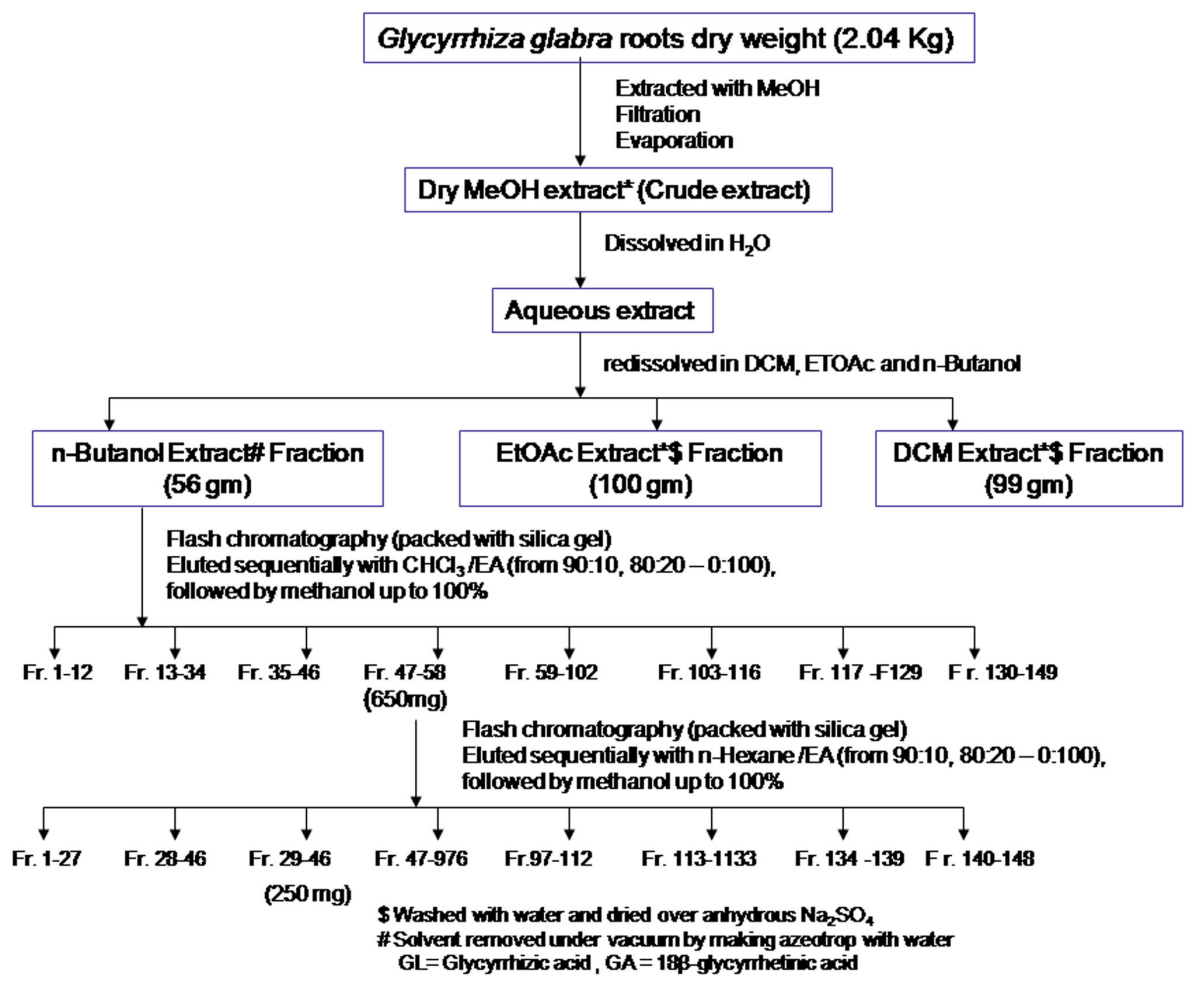
Schematic isolation of GA from *G* *. glabra* roots.

The air dried powdered roots of *G* *. glabra* (2.04 kg) were extracted overnight with methanol (4 x 5L) at room temperature and the combined methanol extract was completely dried under vacuum at 40°C. This dried MeOH extract was dissolved in water and successively fractionated thrice with dichloromethane, ethyl acetate and saturated *n*-butanol, separately. The combined dichloromethane and ethyl acetate extracts were first washed with water and then dried over anhydrous Na _2_SO_4_. Finally, all the three extracts were completely dried under vacuum, which yielded 99.0g of dichloromethane, 100.0g of ethyl acetate and 56.0g of *n*-butanol extracts (Figure 2).

#### 2.1.2: Isolation and Characterization of glycyrrhizic acid (GL)

**Figure 3:**
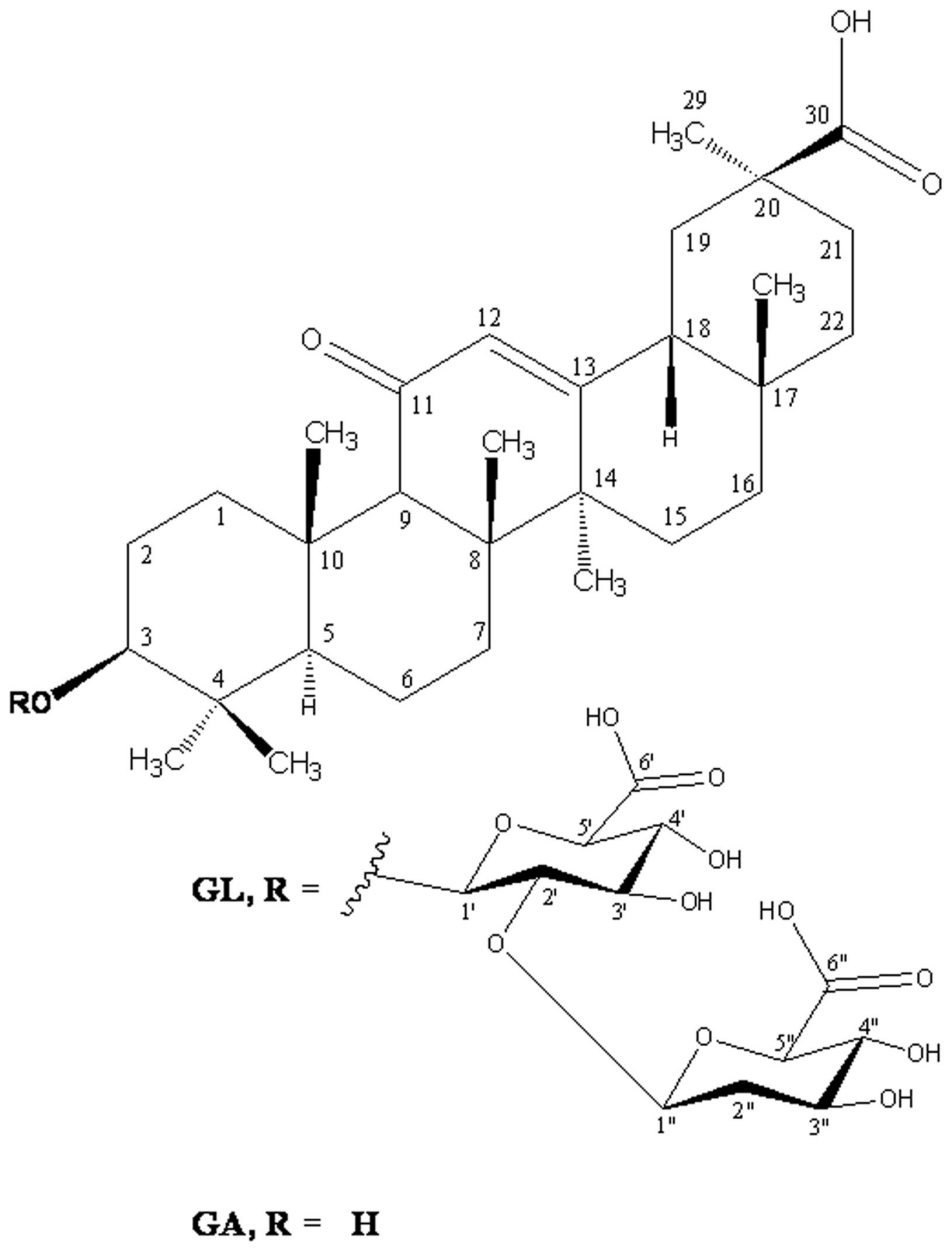
Chemical structures of glycyrrhizic acid (GL) and 18β-glycyrrhetinic acid (GA).

The *n*-butanol extract (1.00 gm) was purified over the flash using Silica gel H (average particle size 10 µM). Gradient elution of the flash was carried out with a mixture of CHCl_3_: MeOH in increasing order (up to 50% MeOH). Fractions of 50ml each were collected and pooled on the basis of their TLC profile. A total of 149 fractions were collected. Fractions 42-58 eluted with CHCl_3_: MeOH (85: 15) afforded a homogeneous compound (650mg), which was characterized as glycyrrhizic acid (GL) on the basis of its ^1^H, ^13^C, DEPT-135 spectroscopic data. C _30_H_46_O_4,_ ESI-MS m/z 823.9 [M+H]^+^, ^1^H NMR (300 MHz, CDCl_3_): Δ 0.76-1.32 (3H each all s, 7 x tert.CH_3_), 2.32 (s, 1 H; 9H), 1.97 (3H, s, C-32), 4.2 (1H, dd, J= 6.8 & 8.7 Hz, 3 α-H), 5.56 (1H, s, H-12), 5.20 (1H,d, H-1’), 3.23 (1H, m, H-2’), 3.62 (1H, m, H 3’ & H-4’), 4.45 (1H, d, H-5’), 12.22 (1H, s, H-6’), 4.86 (1H,d, H-1”), 3.53 (1H, m, H-2”, H-3” & H-4”), 4.5 (1H, d, H-5”), 12.3 (1H, s, H-6”). ^13^C NMR: 39.9 (C-1),27.0 (C-2),88.7 (C-3),37.8 (C-4),55.5 (C-5),18.1 (C-6),33.2 (C-7),43.7 (C-8),62.4 (C-9),37.6 (C-10),199.9 (C-11),128.9 (C-12),169.9 (C-13),45.8 (C-14),28.3 (C-15),26.8 (C-16),32.4 (C-17),48.9 (C-18),41.9 (C-19),44.3 (C-20),31.8 (C-21),38.6 (C-22),28.0 (C-23),16.8 (C-24),17.0 (C-25),19.0 (C-26),23.7 (C-27),28.7 (C-28),28.9 (C-29),179.4 (C-30),104.1 (C-1’),83.1 (C-2’),75.8 (C-3’),71.7 (C-4’),76.5 (C-5’),170.5 (C-6’),105.2 (C-1”),75.4 (C-2”),76.9 (C-3”),71.8 (C-4”), 75.9 (C-5”), 170.6 (C-6”) (Figure 3).

#### 2.1.3: Acid Hydrolysis of glycyrrhizic acid (GL)

Further, GL (650mg) was dissolved in 25 ml of 10% H_2_SO_4_ solution in MeOH and was refluxed for 3-4 hrs. After completion of hydrolysis, the reaction mixture was diluted with water and neutralized with 10% NaOH solution, extracted with CHCl_3_ and evaporated under vacuum till dryness (wt. = 450.0 mg).

#### 2.1.4: Purification of aglycone 18β-glycyrrhetinic acid (GA)

The aglycone (450 mg) was purified over the flash using Silica gel H. A total of 148 fractions were collected and pooled on the basis of their TLC profile. The fractions 29-46 eluted with CHCl_3_: MeOH (99:1) afforded 18β-glycyrrhetinic acid (GA, 250mg); C_30_H_46_O_4,_ ESI-MS m/z 471 [M+H] ^+^, ^1^H NMR (300 MHz, CDCl_3_): Δ 0.76-1.32 (3H each all s, 7 x tert.CH_3_) 2.32 (s, 1 H, 9H), 3.36 (1H, dd, J= 6.8 & 8.5 Hz, 3 α-H) 5.62 (1H, m, H-12). ^13^C NMR: 39.9 (C-1), 27.0 (C-2), 78.1 (C-3), 37.8 (C-4), 55.5 (C-5), 18.1 (C-6), 33.2 (C-7), 43.7 (C-8), 62.4 (C-9), 37.5 (C-10), 199.1 (C-11), 128.9 (C-12), 169.9 (C-13), 45.8 (C-14), 28.3 (C-15), 26.8 (C-16), 32.4 (C-17), 48.9 (C-18), 41.9 (C-19), 44.3 (C-20), 31.8 (C-21), 37.5 (C-22), 27.8 (C-23), 16.8 (C-24), 17.0 (C-25), 19.0 (C-26), 23.7 (C-27), 28.8 (C-28), 28.9 (C-29), 179.4 (C-30) (Figure 3).

### 2.2: *In-vitro, In-silico* and *In-vivo* anti-malarial assay

#### 2.2.1: *In vitro* anti-malarial activity

GA was initially evaluated for anti-malarial activity against *Plasmodium falciparum* (strain NF 54). The parasite was cultured on red blood cells (B+) suspended in RPMI 1640 medium under humidified conditions of 37°C and 5% CO_2_. Before initiation of the assay, parasites were synchronized through 5% sorbitol treatment. GA was dissolved in DMSO and evaluated at three concentrations (100, 10 and 1 μg) in triplicate to derive IC_50_ values. The treatment was given for 24 hours and the parasitaemia from each treatment was microscopically evaluated through Giemsa staining of the blood smears.

#### 2.2.2: *In-silico* molecular docking studies

**Figure 4:**
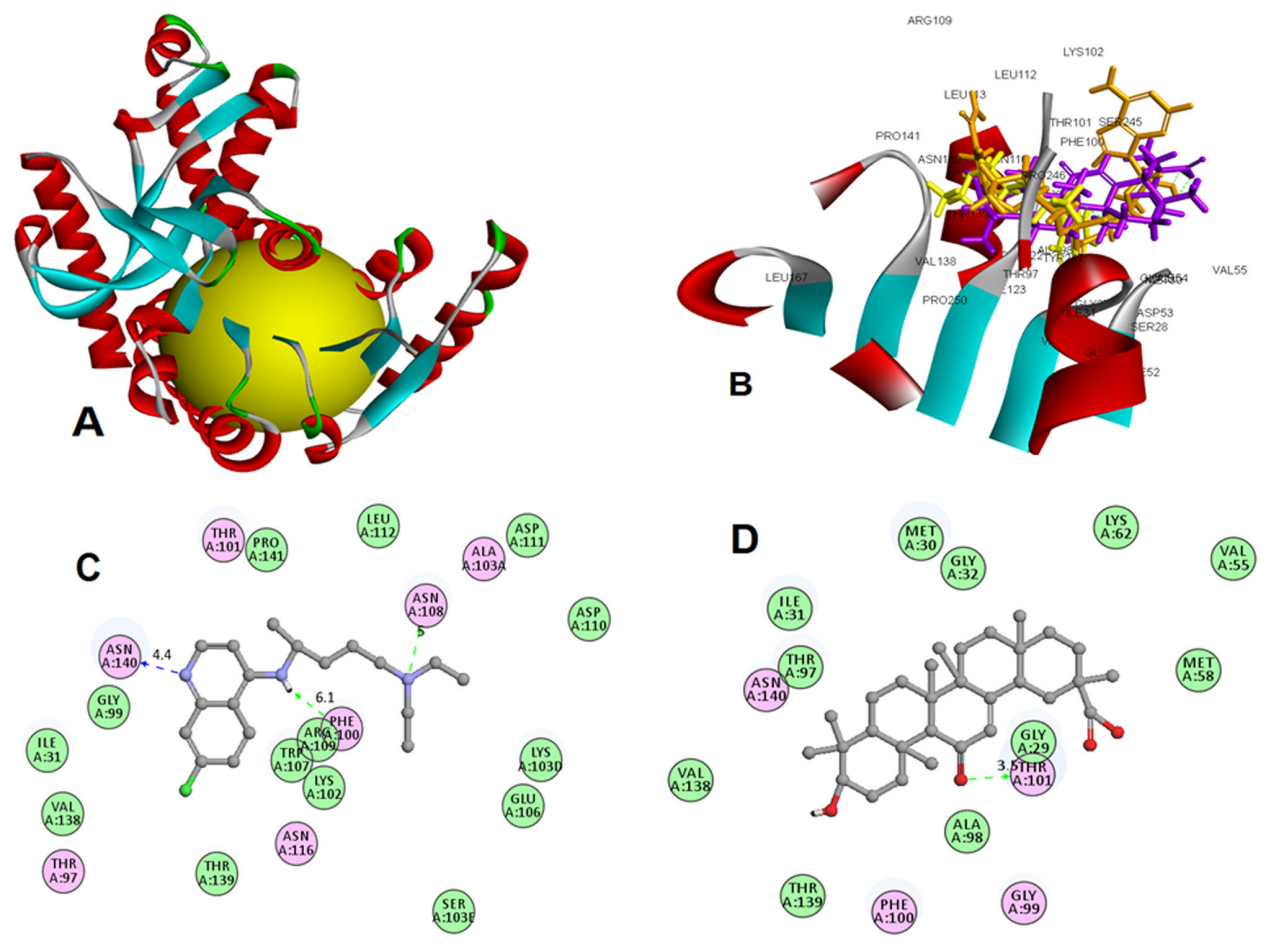
Structural model *Plasmodium falciparum* lactate dehydrogenase (*pf*L*DH*) (*PDB: 1CEQ*) with NADH binding site (yellow color) (A). Superimposition of the best conformation of GA (in purple), CQ (in yellow) and NADH (in orange) in the active site pocket of *P. falciparum* enzyme lactate dehydrogenase *pf*LDH) (B). 2-D diagrams illustrating protein-ligand interactions: (C) Compound CQ; (D) Compound GA.

The molecular docking and visualization studies were performed through Discovery Studio v3.5 (Accelrys Inc., USA, 2013) molecular modeling software. For target protein preparation, structure of *P. falciparum* lactate dehydrogenase (*pf*LDH) (PDB: 1CEQ) [21] was retrieved from a repository of experimentally elucidated crystal structure of biological macromolecules available at the Brookhaven Protein Data Bank (PDB), USA (http://www.rcsb.org/pdb). Initially the protein preparation protocol was used to perform tasks such as inserting missing atoms in incomplete residues, deleting alternate conformations (disorder), removing water, standardizing names of the atoms, modeling missing loop regions and protonating titratable residues. The molecular docking studies were performed to generate the bioactive binding poses of inhibitors in the active site (i.e., NADH binding pocket) of the enzyme by using a LibDock program of Discovery Studio. LibDock uses protein site features referred to as hot spots, consisting of two types (polar and apolar). Then the ligand poses were placed into the polar and apolar receptor interactions site. For energy minimization of the ligands MMFF force field was used under parameterization step. In the present study, the NADH binding pocket was used to define the active site referred as hot spots (Figure 4A). The CAESAR (Conformer Algorithm based on Energy Screening and Recursive build-up) method was used for generating conformations. The Smart Minimiser was used for *in-situ* ligand minimization. All other docking and consequent scoring parameters used were kept at their default settings. The 2-D diagram of docking was derived to identify actual interacting receptor residues with bound ligand.

#### 2.2.3: *In vivo* anti-malarial activity

*P. berghei* K173 (sensitive to CQ at 10 mg/Kg), was used to assess the *in-vivo* intrinsic anti-malarial activity. Parasites were maintained *in-vivo* by a weekly passage in mice as earlier reported [22]. The infection free female Swiss inbred mice, 6-8 weeks old, weighing 18–22g was maintained under standard environmental conditions of 22±3°C, 12:12 dark-to-light cycle with food and water. As reported earlier (Boniface and Pal 2013), mice were infected by intraperitoneal injection of 1×10^6^ infected erythrocytes, diluted in 0.2 ml of sterile acid citrate dextrose (citric acid 7.3g, sodium citrate 22.0g and dextrose 24.5g, dissolved in 1,000 ml of triple distilled water). Parasitaemia was monitored through microscopic examination of Giemsa-stained thin blood smears. Parasitized erythrocytes were obtained from a donor infected mice with 8–10% parasitaemia from the retro orbital plexus in citrate saline (trisodium citrate 2%, NaCl 0.85%) and diluted further so as to contain one million (1×10^6^) parasitized erythrocytes, which was injected intraperitoneally to mice and divided into 5 groups of six mice each. Thin blood smears were drawn from the tip of the tail and stained with standard Giemsa stain. Parasitaemia was determined by counting RBCs comprising of both the parasitized and the normal RBCs. The blood smears were made every alternate day from day 4 till day 28. The results are presented as mean ± SEM. Significance between control and drug treated group were determined using one way analysis of variance (ANOVA) through Instat Software. Tukey’s test was used to compare the means and p < 0.05 was considered significant.

## Results

### 3.1: Isolation and characterization of GA

The air dried powdered roots of *G* *. glabra* were cold extracted with MeOH and the completely dried MeOH extract was successively fractionated with dichloromethane, ethyl acetate and *n*-butanol as per the details given in the experimental section. The *n*-butanol extract so obtained was purified over the flash using Silica gel H (average particle size 10 µM), which afforded a 650mg of homogeneous compound characterized as GL on the basis of its 1D and 2D NMR spectroscopic data [18]. Further, GL was acid hydrolyzed and the aglycone was again purified on flash using Silica gel H, which afforded a 250mg of a pure compound characterized as GA on the basis of its 1D and 2D NMR spectroscopic data [23,24].

### 3.2: *In vitro* activity against *Plasmodium*

The GA was tested against *P. falciparum* NF 54 (*in vitro*) and *P. berghei* K173 (*in vivo*), which were CQ sensitive. When *P. falciparum* was subjected to GA in graded doses, an IC_50_ of 1.69µg/ml was derived as against 0.015µg/ml for CQ.

### 3.3: *In-silico* studies

#### 3.3.1: Docking studies

The docking results are summarized in Table 1, which revealed that GA has moderate docking score (LibDock) of 71.18 for the target protein *pf*LDH in comparison to the standard anti-malarial drug CQ (CQ) i.e., 131.15. Docking of GA on *pf*LDH residues showed single hydrogen (H) bond formation with threonine (THR-101). Similarly, molecular binding of CQ exhibited three hydrogen (H) bonds with phenylalanine (PHE-100) and asparagine (ASN-108, ASN-140). The residues of NADH binding site on *pf*LDH interact with GA and CQ (Table 1). A similar binding site affinity was represented by the superimposition of co-crystallized NADH and docked compounds GA and CQ on PfLDH (Figure 4B). The results of molecular docking and superimposition indicate that the candidate compound GA bound well within the NADH binding pocket of *pf*LDH, but showed slightly lower binding affinity than standard drug CQ. The 2D diagram of molecular docking for compounds CQ (Figure 4C) and GA (Figure 4D) showed interaction of *pf*LDH hydrophobic amino acid residues with bound ligands as represented by different colors e.g., pink indicates electrostatic interaction, purple indicates covalent bond and green color indicates Vander-Waals molecular interaction.

**Table 1:** Details of LibDock score, active site pocket residues and hydrogen bonds revealed through molecular docking of GA, CQ and NADH on Lactate dehydrogenase (PDB: 1CEQ) of *P. falciparum*.

**Target**	**Ligand**	**LibDock score**	**Residues of the NADH binding**	**Residues involved in H-bond**	**No. of H-bond**
pfLDH	NADH	159.04	GLY-27, SER-28, GLY-29, MET-30, ILE-31, GLY-32, ASP-53, VAL-55, THR-97, ALA-98, GLY-99, PHE-100, THR-101, LYS-102, TRP-107, ARG-109, VAL-138, THR-139, ASN-140, PRO-246, TYR-247, PRO-250	GLY-29, MET-30,	7
	(CQ)	131.2	ILE-31, THR-97, GLY-99, PHE-100, THR-101, LYS-102, TRP-107, ARG-109, VAL-138, THR-139, ASN-140	PHE-100, ASN-108, ASN-140,	3
	(GA)	71.18	GLY-29, MET-30, ILE-31, GLY-32, VAL-55, THR-97, ALA-98, GLY-99, PHE-100, THR-101, VAL-138, THR-139, ASN-140	THR-101	1

#### 3.3.2: Bioavailability and drug likeness screening

**Figure 5:**
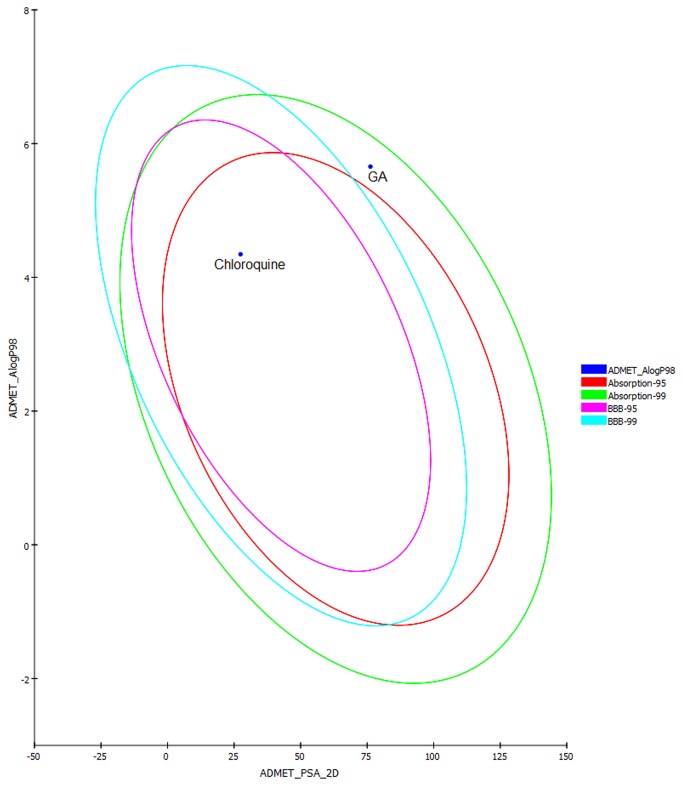
Adsorption model of the candidate compounds.

**Figure 6:**
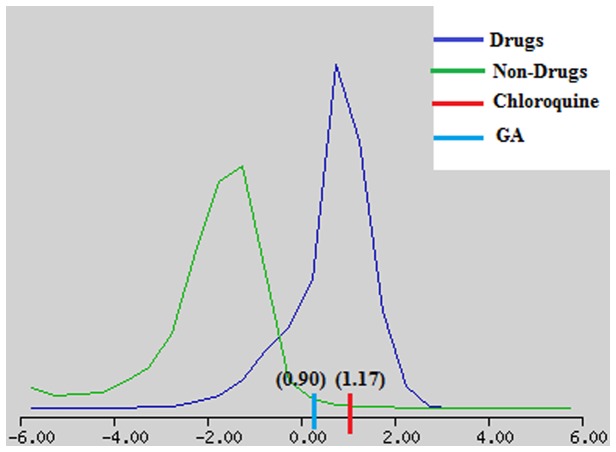
Drug likeness score of 0.9 for GA and 1.17 for CQ lie under the drugs range.

Since docking studies showed promising results, the chemical descriptors for the pharmacokinetic properties were also calculated to check the compliance of GA with standard descriptors. For this aqueous solubility, blood brain barrier penetration, cytochrome P450 2D6 binding, hepatotoxicity, intestinal absorption and plasma protein binding were evaluated through Discovery Studio v3.5 (Accelrys, USA, 2013) molecular modeling software. A biplot (Figure 5) was also provided which shows the two analogous confidence ellipses i.e., 95% and 99% for the blood brain barrier penetration and human intestinal absorption models, respectively. The candidate compound GA showed water soluble nature, moderate intestinal absorption, and binding to plasma protein. Besides, no predictive hepatotoxicity was observed during ADME screening (Table 2). The predicted ADME results of GA were found comparable to standard range. Although drug likeness screening results showed that compound GA violate Lipinski’s rule of five but within acceptable limit, in comparison to the control drug, CQ (Table 3). Moreover, to analyze the drug likeness score, GA was screened against the chemical fingerprints database ‘World Drug Index’ (http://thomsonreuters.com/world-drug-index/). The results showed similar drug likeness score i.e., 0.9 and 1.17 for GA and CQ respectively (Figure 6). Four important toxicity parameters i.e., mutagenicity, tumorigenicity, irritation, and reproductive/developmental toxicity were calculated for GA and CQ through the OSIRIS program (http://www.organic-chemistry.org/prog/peo/) (Table 4).

**Table 2:** Predicted ADME parameters (DS v3.5, Accelrys, USA).

**Compound Name**	**Aqueous solubility**	**Blood brain barrier penetration**	**CYP2D6 Binding**	**Hepatotoxicity**	**Intestinal absorption**	**Plasma protein binding**
CQ	2 (low)	0 (very high)	True (inhibitor)	True (toxic)	0 (good)	True (highly bounded)
GA	1 (very low, but possible)	4 (underfined)	False (non inhibitor)	False (non-toxic)	1 (moderate)	True (highly bounded)

**Table 3:** Compliance of compounds to the theoretical parameters of oral bioavailability and drug likeness properties.

**Compound**	**Pharmacokinetic properties (ADME**)** dependent on chemical descriptors**								
	**ADM**	**AE**	**ADME**	**AD**					**Lipinski’s rule of 5 violation**
	**Oral bioavailability: TPSA (Å2**	**MW**	**Log P**	**H- bond acceptor**			**H-bond acceptor**		
				**NH_2_ group count**	**-NH group count**	**-OH group count**	**-N atom count**	**-O atom count**	
CQ	28.157	319.88	3.808	0	1	0	3	0	0
GA	74.598	470.69	6.101	0	0	1	0	3	1

**Table 4:** Details of computational toxicity risk parameters of GA and CQ calculated by OSIRIS.

**Compound**	**Toxicity risk parameters**			
	**MUT**	**TUMO**	**IRRI**	**REP**
CQ	No risk	No risk	No risk	No risk
GA	No risk	No risk	No risk	No risk

Note: MUT= Mutagenicity, TUMO= Tumorogenicity, IRRI= Irritation, REP= reproductively effective

### 3.4: *In vivo activity against Plasmodium*

On the basis of *in-vitro* and *in-silico* results, GA was further evaluated in mice infected with *P. berghei*, which showed a dose dependent activity (6.68±2.19, 1.49±1.04 and 0±0% parasitemia at 62.5, 125 & 250mg/kg respectively) as against 20.57±3.13% parasitemia in infected but non-treated animals.

## Discussion

### 4.1: Isolation and characterization of GA

The GA was isolated and discussed in the experimental section and characterized on the basis of their 1D and 2D NMR spectroscopic data which were in full agreement with the 1D and 2D NMR spectroscopic data recorded for an authentic sample of 18β-glycyrrhetinic acid (ACROS ORGANICS).

### 4.2: Molecular interaction through docking

Recent studies have paved the way for identifying valuable leads [9], which could be useful for anti-malarial aspects as there is a persistent urge for novel compounds that can act at therapeutic targets. *P. falciparum* lactate dehydrogenase enzyme (*pf*LDH) represents one of such potential molecular target. Earlier studies (Read et al., 1999) suggest that CQ does not directly inhibit the enzyme but it competes with NADH for binding with the enzyme. Molecular docking results in the present study also revealed the similar molecular interaction and showed that bound compound GA interact with amino acid residues e.g., GLY-29, MET-30, ILE-31, GLY-32, VAL-55, THR-97, ALA-98, GLY-99, PHE-100, THR-101, VAL-138, THR-139, ASN-140, similar to NADH on the target enzyme *pf*LDH. In silico docking studies suggest that GA has similar binding site pocket residues as shown by NADH binding site on *pf*LDH, therefore suggesting a competitive inhibition similar to CQ, which resulted stability and potent activity.

### 4.3: Compliance with Bioavailability and ADMET parameters

The ADMET is one of the key measures which influence the drug levels and kinetics of drug and influence the pharmacological activity of the compound as a drug. In this study the aqueous solubility prediction (defined in water at 25°C) showed that GA is slightly less soluble. LogP value, which is a measure of lipophilicity and is the ratio of the solubility of the compound in octanol compared to its solubility in water was found to be quite high for GA in comparison to CQ, implicating low oral bioavailability (Table 2). Apart from the above, excretion process that eliminates the compound from human body also depends on LogP. The GA showed high (>= 90%) binding with the carrier proteins in the blood which depicts the efficiency of a drug. The standard drug CQ showed hepatotoxicity, while studied compound GA indicates no such toxicity (Table 2). It is important that the drugs which are administered orally must get absorbed in the intestine. In this regard, the predicted results showed that both the compounds can get easily absorbed from the intestine, as referred by the chemical descriptor TPSA. The topological polar surface area (TPSA) was calculated as a chemical descriptor for passive molecular transport through membranes. The results exhibited a high TPSA value for GA in comparison to CQ but within acceptable limit i.e. <140 Å^2^ (Table 3). Results of overall drug-likeness score against the chemical fingerprints database (World Drug Index database) showed that both GA and CQ fall within the standard range of drugs (Figure 6). Similarly, results of toxicity risk assessment screening at high doses or long term use, also suggest compliance of compound GA with standard drug CQ. The toxicity screening results showed that GA competes well with known drug CQ and indicates no toxicity (Table 4).

### 4.4: Anti-malarial activity of GA

The *in vitro* anti-malarial activity of GA against *P. falciparum* was comparatively lower than the standard drug CQ, This was also correlated with the docking studies which also revealed a moderate docking score for GA when compared to CQ. Further, *in vivo* studies on GA showed a dose dependent activity when compared to infected but non-treated animals, though the cure rate was lower than the standard drug CQ. The likely reason for a comparative lower activity may be due to the low oral bioavailability of GA due to its higher logP value and low water solubility. GA being a natural compound has the possibility of further exploration and lead optimization by QSAR approach in resistant strains of *Plasmodium.* The triterpenoids from plants earlier showed *in-vitro* anti-plasmodial activity were rarely in-vivo evaluated for their anti-malarial potential [25,26].

## Conclusion

In view of potential anti-malarial activity, non-toxicity to the host, favourable pharmacokinetics and drug-like properties, GA was identified as a lead candidate for further detailed *in-silico*, biological and pharmaceutical investigations. This is the first ever report of GA showing *in-vitro, in-silico* and *in vivo* anti-malarial activity. Further, the effect of GA on the innate immune system in malaria infected mice and combination studies of GA with established anti-malarials can be carried out against the drug resistant strains to increase or revoke the efficacy of the resistant anti-malarial drugs. It is thus concluded that, the discovery of the novel lead molecule might hopefully bring advancement in the safe and effective treatment of malaria. Further, QSAR and docking guided anti-malarial lead optimization is under progress, which will assist in elucidating the precise mechanism of action.

## References

[1] Daily JP (2006) Antimalarial drug therapy: the role of parasite biology and drug resistance. J Clin Pharmacol 46: 1487-1497. doi:10.1177/0091270006294276. PubMed: 17101748. 17101748 10.1177/0091270006294276

[2] W.H.O. (2012) orld Malaria Report 2012. Available at: http://www.who.int/malaria/publications/world_malaria_report_2012/report/en/wmr2012_full_report.pdf. Accessed: August /2013.

[3] Penna-Coutinho J, Cortopassi WA, Oliveira AA, França TC, Krettli AU (2011) Antimalarial activity of potential inhibitors of Plasmodium falciparum lactate dehydrogenase enzyme selected by docking studies. PLOS ONE 6: e21237. doi:10.1371/journal.pone.0021237. PubMed: 21779323. 21779323 10.1371/journal.pone.0021237 PMC3136448

[4] Gardner MJ, Hall N, Fung E, White O, Berriman M et al. (2002) Genome sequence of the human malaria parasite Plasmodium falciparum. Nature 419: 498–511. doi:10.1038/nature01097. PubMed: 12368864. 12368864 10.1038/nature01097 PMC3836256

[5] Takenaka T (2001) Classical vs reverse pharmacology in drug discovery. BJU Int 88 (Suppl 2): 7-10; discussion 49-50 doi:10.1111/j.1464-410X.2001.00112.x. PubMed: 11589663. 11589663 10.1111/j.1464-410x.2001.00112.x

[6] Sessions RB, Dewar V, Clarke AR, Holbrook JJ (1997) A model of Plasmodium falciparum lactate dehydrogenase and its implications for the design of improved antimalarials and the enhanced detection of parasitaemia. Protein Eng 10: 301-306. doi:10.1093/protein/10.4.301. PubMed: 9194154. 9194154 10.1093/protein/10.4.301

[7] Harwood JL (1996) Recent advances in the biosynthesis of plant fatty acids. Biochim Biophys Acta 1301: 7–56. doi:10.1016/0005-2760(95)00242-1. PubMed: 8652653. 8652653 10.1016/0005-2760(95)00242-1

[8] Roy KK, Bhunia SS, Saxena AK (2011) CoMFA, CoMSIA, and Docking Studies on Thiolactone-Class of Potent Anti-malarials: Identification of Essential Structural Features Modulating Anti-malarial Activity. Chem Biol Drugs Des 78: 483–493. doi:10.1111/j.1747-0285.2011.01158.x. PubMed: 21672165. 10.1111/j.1747-0285.2011.01158.x 21672165

[9] Qidwai T, Yadav DK, Khan F, Dhawan S, Bhakuni RS (2012) QSAR, Docking and ADMET Studies of Artemisinin Derivatives for Antimalarial Activity Targeting Plasmepsin II, a Hemoglobin-Degrading Enzyme from P. falciparum. Curr Pharm Des 18: 6133-6154. doi:10.2174/138161212803582397. PubMed: 22670592. 22670592 10.2174/138161212803582397

[10] Sen D, Chatterjee TK (2013) Pharmacophore modeling and 3D quantitative structure-activity relationship analysis of febrifugine analogues as potent antimalarial agent. J Adv Pharm Technol Res 4: 50–60. PubMed: 23662282. 23662282 10.4103/2231-4040.107501 PMC3645363

[11] Srivastava V, Negi AS, Kumar JK, Gupta MM, Khanuja SP (2005) Plant-based anticancer molecules: a chemical and biological profile of some important leads. Bioorg Med Chem Lett 13: 5892-5908. doi:10.1016/j.bmc.2005.05.066. PubMed: 16129603. 10.1016/j.bmc.2005.05.066 16129603

[12] Gautam R, Jachak SM (2009) Recent developments in anti-inflammatory natural products. Med Res Rev 29: 767-820. doi:10.1002/med.20156. PubMed: 19378317. 19378317 10.1002/med.20156

[13] Gao D, Tang S, Tong Q (2012) Oleanolic acid liposomes with polyethylene glycol modification: promising antitumor drug delivery. Int J Nanomed 7: 3517-3526. doi:10.2147/IJN.S31725. PubMed: 22848175. 10.2147/IJN.S31725 PMC3405888 22848175

[14] Liu J (1995) Pharmacology of oleanolic acid and ursolic acid. J Ethnopharmacol 49: 57-68. doi:10.1016/0378-8741(95)90032-2. PubMed: 8847885. 8847885 10.1016/0378-8741(95)90032-2

[15] Singh GB, Singh S, Bani S, Gupta BD, Banerjee SK (2011) Anti-inflammatory activity of oleanolic acid in rats and mice. J Pharm Pharmacol 44: 456-458. PubMed: 1359067. 10.1111/j.2042-7158.1992.tb03646.x 1359067

[16] Zhu YM, Shen JK, Wang HK, Cosentino LM, Lee KH (2001) Synthesis and anti-HIV activity of oleanolic acid derivatives. Bioorg Med Chem Lett 11: 3115-3118. doi:10.1016/S0960-894X(01)00647-3. PubMed: 11720855. 11720855 10.1016/s0960-894x(01)00647-3

[17] Cimanga RK, Tona GL, Mesia GK, Kambu OK, Bakana DP et al. (2006) Guided Isolation of Antimalarial Triterpenoid Acids from the Leaves of Morinda lucida. Pharm Biol 44: 677-681. doi:10.1080/13880200601009123.

[18] Kalani K, Kushwaha V, Verma R, Murthy PK, Srivastava SK (2013) Glycyrrhetinic acid and its analogs: A new class of antifilarial agents. Bioorg Med Chem Lett 23: 2566-2570. doi:10.1016/j.bmcl.2013.02.115. PubMed: 23541646. 23541646 10.1016/j.bmcl.2013.02.115

[19] Assessment report on Glycyrrhiza glabra L. and/or Glycyrrhiza inflata Bat. and/or Glycyrrhiza uralensis Fisch., radix, Committee on Herbal Medicinal Products (HMPC), EMA/HMPC/571122/2010 Corr.European Medicines Agency, Science Medicines. Health (2013). Available: http://www.ema.europa.eu/docs/en_GB/document_library/Herbal_-_HMPC_assessment_report/2012/08/WC500131285.pdf. Accessed: august/2013

[20] Khare CP (2004) Encyclopedia of Indian Medicinal Plants. New York: Springer-Verlag. 233pp.

[21] Read JA, Wilkinson KW, Tranter R, Sessions RB, Brady RL (1999) Chloroquine binds in the cofactor binding site of Plasmodium falciparum lactate dehydrogenase. J Biol Chem 274: 10213-10218. doi:10.1074/jbc.274.15.10213. PubMed: 10187806. 10187806

[22] Boniface PK, Pal A (2013) Substantiation of the ethnopharmacological use of Conyza sumatrensis (Retz.) E.H.Walker in the treatment of malaria through in-vivo evaluation in Plasmodium berghei infected mice. J Ethnopharmacol 145: 373-377. doi:10.1016/j.jep.2012.10.025. PubMed: 23123263. 23123263 10.1016/j.jep.2012.10.025

[23] Macías A, Pelaz S, Morata G (1994) Genetic factors controlling the expression of the abdominal-A gene of Drosophila within its domain. Mech Dev 46: 15-25. doi:10.1016/0925-4773(94)90034-5. PubMed: 7915130. 7915130 10.1016/0925-4773(94)90034-5

[24] Nick A, Rali T, Sticher O (1995) Biological screening of traditional medicinal plants from Papua New Guinea. J Ethnopharmacol 49: 147-156. doi:10.1016/0378-8741(95)01315-6. PubMed: 8824740. 8824740 10.1016/0378-8741(95)01315-6

[25] Ramalhete C, Lopes D, Mulhovo S, Molnár J, Rosário VE et al. (2010) New antimalarials with a triterpenic scaffold from Momordica balsamina. Bioorg Med Chem 18: 5254-5260. doi:10.1016/j.bmc.2010.05.054. PubMed: 20541427. 20541427 10.1016/j.bmc.2010.05.054

[26] Ramalhete C, da Cruz FP, Lopes D, Mulhovo S, Rosário VE et al. (2011) Triterpenoids as inhibitors of erythrocytic and liver stages of Plasmodium infections. Bioorg Med Chem 19: 7474-7481. doi:10.1016/j.bmc.2011.10.044. PubMed: 22071523. 22071523 10.1016/j.bmc.2011.10.044

